# 3-Methyl-5α-cholest-2-ene

**DOI:** 10.1107/S160053681003117X

**Published:** 2010-08-11

**Authors:** Kamal Aziz Ketuly, A. Hamid A. Hadi, Seik Weng Ng, Edward R. T. Tiekink

**Affiliations:** a Department of Chemistry, University of Malaya, 50603 Kuala Lumpur, Malaysia

## Abstract

In the title cholestane derivative, C_28_H_48_ [systematic name: (1*S*,2*S*,7*R*,10*R*,11*R*,14*R*,15*R*)-2,5,10,15-tetra­methyl-14-[(2*R*)-6-methyl­heptan-2-yl]tetra­cyclo­[8.7.0.0^2,7^.0^11,15^]hepta­dec-4-ene], the cyclo­hexene ring adopts a half-chair conformation. The parent 5α-cholest-2-ene and the equivalent fragment of the title compound are almost superimposable (r.m.s. deviation = 0.033 Å).

## Related literature

For background to this study, see: Ketuly & Hadi (2010 ▶). For the synthesis, see: Barton *et al.* (1956 ▶). For a discussion of the structural features of cholestane derivatives, see: Rajnikant *et al.* (2006 ▶). For the structure of 5α-cholest-2-ene, see: Kemlo *et al.* (1979 ▶). For ring conformational analysis, see: Cremer & Pople (1975 ▶).
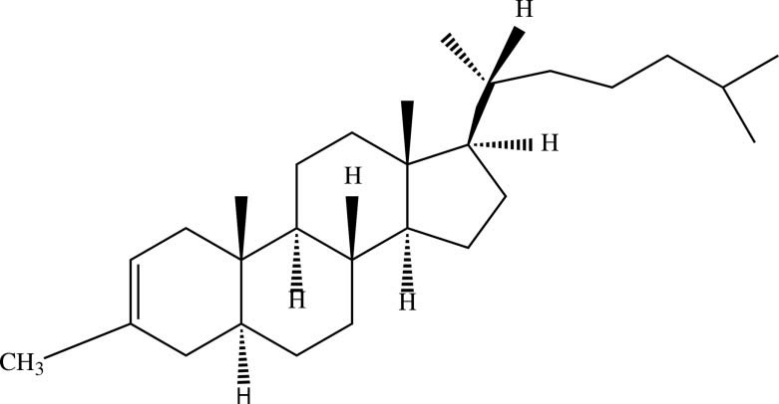


## Experimental

### 

#### Crystal data

C_28_H_48_
*M* *_r_* = 384.66
Monoclinic, 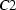

*a* = 22.216 (3) Å
*b* = 11.7576 (15) Å
*c* = 9.6335 (13) Å
β = 106.652 (2)°
*V* = 2410.9 (5) Å^3^
*Z* = 4
Mo *K*α radiation
μ = 0.06 mm^−1^
*T* = 100 K
0.35 × 0.15 × 0.05 mm

#### Data collection

Bruker SMART APEX CCD diffractometer
Absorption correction: multi-scan (*SADABS*; Sheldrick, 1996 ▶) *T*_min_ = 0.786, *T*_max_ = 0.862
11615 measured reflections
2902 independent reflections
2379 reflections with *I* > 2σ(*I*)
*R*_int_ = 0.060

#### Refinement

*R*[*F* ^2^ > 2σ(*F* ^2^)] = 0.047
*wR*(*F* ^2^) = 0.121
*S* = 1.02
2902 reflections
253 parameters
1 restraint
H-atom parameters constrained
Δρ_max_ = 0.24 e Å^−3^
Δρ_min_ = −0.20 e Å^−3^

### 

Data collection: *APEX2* (Bruker, 2009 ▶); cell refinement: *SAINT* (Bruker, 2009 ▶); data reduction: *SAINT*; program(s) used to solve structure: *SHELXS97* (Sheldrick, 2008 ▶); program(s) used to refine structure: *SHELXL97* (Sheldrick, 2008 ▶); molecular graphics: *ORTEP-3* (Farrugia, 1997 ▶), *DIAMOND* (Brandenburg, 2006 ▶) and Qmol (Gans & Shalloway, 2001 ▶); software used to prepare material for publication: *publCIF* (Westrip, 2010 ▶).

## Supplementary Material

Crystal structure: contains datablocks global, I. DOI: 10.1107/S160053681003117X/hb5593sup1.cif

Structure factors: contains datablocks I. DOI: 10.1107/S160053681003117X/hb5593Isup2.hkl

Additional supplementary materials:  crystallographic information; 3D view; checkCIF report

## Figures and Tables

**Fig. 1.**
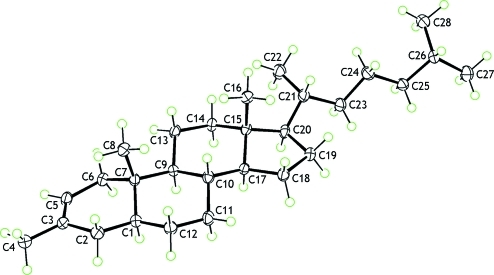
The molecular structure of (I) showing displacement ellipsoids at the 50% probability level.

**Fig. 2.**
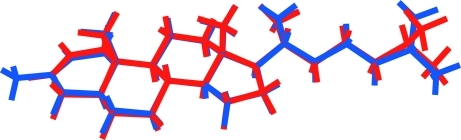
Overlay diagram of (I), shown in blue, with the parent 5α-cholest-2-ene, shown in red.

## supplementary crystallographic information

### Comment

The title compound, 3-methyl-5α-cholest-2-ene, (I), has been prepared previously (Barton *et al.*, 1956) as a precursor for the synthesis of steroidal boronic acids and boronates (Ketuly *et al.*, 2010). The geometric and structural features for a series of cholestane derivatives has been described (Rajnikant *et al.*, 2006).

In the structure of (I), Fig. 1, the cyclohexene has a half-chair conformation: the ring-puckering parameters are q_2_ = 0.387 (3) Å, q_3_ = -0.318 (3) Å, QT = 0.501 (3) Å, φ_2_ = 149.2 (5) ° (Cremer & Pople, 1975). With the exception of a small difference in orientation of the terminal residues, the structure of (I) is virtually super-imposable upon the structure of the parent 5α-cholest-2-ene structure (Kemlo *et al.*, 1979). The r.m.s. deviation between the two molecules is 0.033 Å (Gans & Shalloway, 2001).

### Experimental

The synthesis of the title compound is a modified version of the literature procedure (Barton *et al.*, 1956). 5α-cholestan-3-one (3 g, 8 mmol) in dry ether (40 ml) was added to methylmagnesium iodide [prepared by by the gradual addition of methyl iodide (1.2 ml, 20 mmol) in dry ether (15 ml) to magnesium (0.5 g, 21 mmol) in dry ether (10 ml) during 30 min. with continuous stirring and cooling] and the resulting solution was refluxed for 3 h. The reaction mixture was poured on to ice, then a solution of H_2_SO_4_ (1.52 g) and water (10 ml) added with stirring. The mixture was extracted three times with ether and washed with saturated NaHCO_3_, then with water until neutral. Extracts were dried and evaporated, yielding a white crystalline product (2.92 g, 94% of the mixture isomers: 3α-hydroxy-3β-methyl-5α-cholestane and 3β-hydroxy-3α-methyl-5α-cholestane, m.p. 368–373 K. The mixture of these stereoisomeric alchohols (2 g) was dissolved in glacial acetic acid (25 ml) on warming, then perchloric acid (72%, *w*/*w*, 10 drops) was added and the solution heated in hot bath (253–263 K) for 30 min. The solvent and excess reagent were evaporated azeotropically. The yellow solid residue was dissolved in ether, washed with NaHCO_3_ then with water, until neutral; each washing was back-extracted three times with ether. The extracts were combined; dried and evaporated. A yellow oily residue, which gradually crystallized was recovered (1.81 g, 95%), mp. 342–346 K. This was recrystallized from ether-MeOH, giving (1.739 g, 91%) of the title compound, mp. 348–350 K. This was further recrystallized and colourless blocks of (I) were grown from carbon ethanol:ether (8:1,*v*/*v*), m.p. 354–355 K (Lit. Barton *et al.* (1956) 356–357 K from light petroleum).

### Refinement

Carbon-bound H-atoms were placed in calculated positions (C—H 0.95 to 1.00 Å) and were included in the refinement in the riding model approximation, with *U*_iso_(H) set to 1.2 to 1.5*U*_equiv_(C). In the absence of significant anomalous scattering effects, 2599 Friedel pairs were averaged in the final refinement. However, the absolute configuration was assigned on the basis of the known chiralty of the 5α-cholestan-3-one starting material (C1 S, C7 S, C9 S, C10 S, C15 R, C17 S, C20 R, C21 R)

### Crystal data

**Table:**

C_28_H_48_	*F*(000) = 864
*M**_r_* = 384.66	*D*_x_ = 1.060 Mg m^−^^3^
Monoclinic, *C*2	Mo *K*α radiation, λ = 0.71073 Å
Hall symbol: C 2y	Cell parameters from 1712 reflections
*a* = 22.216 (3) Å	θ = 2.5–22.9°
*b* = 11.7576 (15) Å	µ = 0.06 mm^−^^1^
*c* = 9.6335 (13) Å	*T* = 100 K
β = 106.652 (2)°	Prism, colourless
*V* = 2410.9 (5) Å^3^	0.35 × 0.15 × 0.05 mm
*Z* = 4	

### Data collection

**Table:**

Bruker SMART APEX CCD diffractometer	2902 independent reflections
Radiation source: fine-focus sealed tube	2379 reflections with *I* > 2σ(*I*)
graphite	*R*_int_ = 0.060
ω scan	θ_max_ = 27.5°, θ_min_ = 1.9°
Absorption correction: multi-scan (*SADABS*; Sheldrick, 1996)	*h* = −28→28
*T*_min_ = 0.786, *T*_max_ = 0.862	*k* = −15→15
11615 measured reflections	*l* = −12→12

### Refinement

**Table:**

Refinement on *F*^2^	Primary atom site location: structure-invariant direct methods
Least-squares matrix: full	Secondary atom site location: difference Fourier map
*R*[*F*^2^ > 2σ(*F*^2^)] = 0.047	Hydrogen site location: inferred from neighbouring sites
*wR*(*F*^2^) = 0.121	H-atom parameters constrained
*S* = 1.02	*w* = 1/[σ^2^(*F*_o_^2^) + (0.0616*P*)^2^ + 0.7202*P*] where *P* = (*F*_o_^2^ + 2*F*_c_^2^)/3
2902 reflections	(Δ/σ)_max_ = 0.001
253 parameters	Δρ_max_ = 0.24 e Å^−^^3^
1 restraint	Δρ_min_ = −0.20 e Å^−^^3^

### Special details

**Table:**

Geometry. All e.s.d.'s (except the e.s.d. in the dihedral angle between two l.s. planes) are estimated using the full covariance matrix. The cell e.s.d.'s are taken into account individually in the estimation of e.s.d.'s in distances, angles and torsion angles; correlations between e.s.d.'s in cell parameters are only used when they are defined by crystal symmetry. An approximate (isotropic) treatment of cell e.s.d.'s is used for estimating e.s.d.'s involving l.s. planes.
Refinement. Refinement of *F*^2^ against ALL reflections. The weighted *R*-factor *wR* and goodness of fit *S* are based on *F*^2^, conventional *R*-factors *R* are based on *F*, with *F* set to zero for negative *F*^2^. The threshold expression of *F*^2^ > σ(*F*^2^) is used only for calculating *R*-factors(gt) *etc*. and is not relevant to the choice of reflections for refinement. *R*-factors based on *F*^2^ are statistically about twice as large as those based on *F*, and *R*- factors based on ALL data will be even larger.

### Fractional atomic coordinates and isotropic or equivalent isotropic displacement parameters (Å^2^)

**Table:**

	*x*	*y*	*z*	*U*_iso_*/*U*_eq_	
C1	0.25641 (12)	0.4995 (2)	0.1799 (3)	0.0211 (6)	
H1A	0.2370	0.5179	0.0752	0.025*	
C2	0.22521 (14)	0.3904 (3)	0.2106 (3)	0.0259 (6)	
H2A	0.2276	0.3322	0.1382	0.031*	
H2B	0.2488	0.3614	0.3074	0.031*	
C3	0.15797 (13)	0.4068 (3)	0.2063 (3)	0.0226 (6)	
C4	0.12007 (14)	0.3006 (3)	0.2017 (3)	0.0289 (7)	
H4A	0.0771	0.3210	0.2002	0.043*	
H4B	0.1392	0.2543	0.2876	0.043*	
H4C	0.1191	0.2572	0.1142	0.043*	
C5	0.13404 (13)	0.5112 (3)	0.2097 (3)	0.0241 (6)	
H5A	0.0909	0.5172	0.2052	0.029*	
C6	0.17105 (12)	0.6191 (3)	0.2201 (3)	0.0226 (6)	
H6A	0.1598	0.6704	0.2902	0.027*	
H6B	0.1588	0.6572	0.1245	0.027*	
C7	0.24280 (12)	0.6016 (2)	0.2674 (3)	0.0178 (5)	
C8	0.26358 (13)	0.5758 (3)	0.4308 (3)	0.0238 (6)	
H8A	0.2421	0.5073	0.4498	0.036*	
H8B	0.2527	0.6402	0.4837	0.036*	
H8C	0.3091	0.5636	0.4631	0.036*	
C9	0.27602 (13)	0.7082 (2)	0.2307 (3)	0.0175 (6)	
H9A	0.2565	0.7224	0.1250	0.021*	
C10	0.34646 (12)	0.6901 (2)	0.2494 (3)	0.0181 (6)	
H10A	0.3679	0.6750	0.3541	0.022*	
C11	0.35657 (12)	0.5880 (2)	0.1617 (3)	0.0233 (6)	
H11A	0.3387	0.6045	0.0572	0.028*	
H11B	0.4022	0.5747	0.1804	0.028*	
C12	0.32603 (13)	0.4813 (2)	0.1997 (3)	0.0224 (6)	
H12A	0.3316	0.4178	0.1369	0.027*	
H12B	0.3470	0.4600	0.3015	0.027*	
C13	0.26474 (12)	0.8173 (2)	0.3085 (3)	0.0204 (6)	
H13A	0.2189	0.8302	0.2868	0.025*	
H13B	0.2819	0.8064	0.4144	0.025*	
C14	0.29511 (12)	0.9230 (2)	0.2637 (3)	0.0193 (6)	
H14A	0.2748	0.9393	0.1600	0.023*	
H14B	0.2883	0.9892	0.3207	0.023*	
C15	0.36558 (12)	0.9056 (2)	0.2883 (3)	0.0173 (6)	
C16	0.39946 (13)	0.8959 (3)	0.4510 (3)	0.0222 (6)	
H16A	0.4446	0.8853	0.4651	0.033*	
H16B	0.3828	0.8307	0.4915	0.033*	
H16C	0.3926	0.9656	0.5003	0.033*	
C17	0.37381 (12)	0.7980 (2)	0.2051 (3)	0.0169 (5)	
H17A	0.3499	0.8119	0.1016	0.020*	
C18	0.44307 (12)	0.7989 (2)	0.2104 (3)	0.0210 (6)	
H18A	0.4504	0.7547	0.1292	0.025*	
H18B	0.4696	0.7676	0.3031	0.025*	
C19	0.45632 (13)	0.9275 (2)	0.1962 (3)	0.0217 (6)	
H19A	0.4954	0.9500	0.2705	0.026*	
H19B	0.4614	0.9437	0.0993	0.026*	
C20	0.39944 (12)	0.9943 (2)	0.2177 (3)	0.0189 (6)	
H20A	0.3708	1.0120	0.1193	0.023*	
C21	0.41998 (12)	1.1083 (3)	0.2951 (3)	0.0216 (6)	
H21A	0.4519	1.0910	0.3894	0.026*	
C22	0.36660 (14)	1.1740 (3)	0.3294 (3)	0.0284 (7)	
H22A	0.3830	1.2450	0.3792	0.043*	
H22B	0.3485	1.1276	0.3919	0.043*	
H22C	0.3341	1.1914	0.2391	0.043*	
C23	0.45225 (13)	1.1824 (3)	0.2065 (3)	0.0228 (6)	
H23A	0.4762	1.1323	0.1590	0.027*	
H23B	0.4194	1.2209	0.1291	0.027*	
C24	0.49675 (14)	1.2726 (3)	0.2937 (3)	0.0263 (7)	
H24A	0.4731	1.3236	0.3410	0.032*	
H24B	0.5301	1.2349	0.3708	0.032*	
C25	0.52703 (13)	1.3430 (2)	0.1990 (3)	0.0216 (6)	
H25A	0.4936	1.3870	0.1297	0.026*	
H25B	0.5452	1.2902	0.1418	0.026*	
C26	0.57833 (12)	1.4256 (2)	0.2788 (3)	0.0205 (6)	
H26A	0.6114	1.3814	0.3508	0.025*	
C27	0.60833 (15)	1.4801 (3)	0.1707 (3)	0.0307 (7)	
H27A	0.6407	1.5342	0.2215	0.046*	
H27B	0.5761	1.5201	0.0958	0.046*	
H27C	0.6274	1.4208	0.1256	0.046*	
C28	0.55335 (14)	1.5163 (3)	0.3599 (3)	0.0330 (7)	
H28A	0.5876	1.5674	0.4094	0.049*	
H28B	0.5359	1.4800	0.4315	0.049*	
H28C	0.5204	1.5600	0.2913	0.049*	

### Atomic displacement parameters (Å^2^)

**Table:**

	*U*^11^	*U*^22^	*U*^33^	*U*^12^	*U*^13^	*U*^23^
C1	0.0200 (13)	0.0226 (15)	0.0225 (13)	−0.0045 (12)	0.0088 (10)	−0.0041 (12)
C2	0.0297 (15)	0.0215 (15)	0.0303 (15)	−0.0038 (12)	0.0146 (12)	−0.0040 (12)
C3	0.0224 (14)	0.0284 (16)	0.0189 (13)	−0.0070 (12)	0.0090 (11)	−0.0049 (12)
C4	0.0311 (16)	0.0308 (17)	0.0285 (15)	−0.0091 (14)	0.0144 (12)	−0.0083 (14)
C5	0.0181 (13)	0.0316 (17)	0.0249 (14)	−0.0042 (12)	0.0099 (11)	−0.0009 (13)
C6	0.0202 (13)	0.0249 (15)	0.0259 (14)	−0.0031 (12)	0.0115 (11)	−0.0030 (12)
C7	0.0167 (12)	0.0183 (13)	0.0192 (13)	−0.0028 (11)	0.0065 (10)	0.0008 (11)
C8	0.0279 (14)	0.0253 (16)	0.0203 (13)	−0.0025 (12)	0.0102 (11)	0.0007 (12)
C9	0.0174 (13)	0.0186 (14)	0.0171 (13)	−0.0036 (11)	0.0062 (10)	−0.0018 (10)
C10	0.0159 (13)	0.0204 (14)	0.0179 (13)	−0.0001 (11)	0.0045 (10)	0.0020 (11)
C11	0.0163 (12)	0.0236 (15)	0.0318 (15)	−0.0010 (12)	0.0096 (11)	−0.0028 (12)
C12	0.0206 (14)	0.0178 (14)	0.0302 (15)	0.0008 (11)	0.0094 (11)	−0.0017 (12)
C13	0.0167 (13)	0.0226 (15)	0.0249 (14)	0.0005 (11)	0.0106 (11)	0.0000 (12)
C14	0.0164 (13)	0.0207 (14)	0.0223 (13)	−0.0016 (11)	0.0080 (10)	0.0012 (11)
C15	0.0172 (13)	0.0191 (15)	0.0171 (12)	−0.0005 (11)	0.0070 (10)	0.0026 (11)
C16	0.0241 (14)	0.0261 (15)	0.0175 (12)	−0.0035 (12)	0.0078 (11)	−0.0004 (11)
C17	0.0143 (12)	0.0209 (14)	0.0162 (12)	−0.0014 (11)	0.0056 (9)	−0.0010 (11)
C18	0.0182 (13)	0.0207 (14)	0.0259 (14)	−0.0011 (12)	0.0092 (11)	−0.0012 (12)
C19	0.0199 (14)	0.0230 (14)	0.0246 (14)	−0.0043 (12)	0.0103 (11)	−0.0012 (11)
C20	0.0168 (12)	0.0236 (14)	0.0169 (12)	−0.0023 (12)	0.0060 (10)	0.0046 (11)
C21	0.0207 (13)	0.0253 (15)	0.0200 (13)	−0.0038 (12)	0.0078 (11)	0.0016 (11)
C22	0.0275 (15)	0.0223 (15)	0.0384 (17)	−0.0014 (13)	0.0143 (13)	−0.0020 (13)
C23	0.0249 (15)	0.0220 (15)	0.0223 (14)	−0.0047 (12)	0.0081 (11)	0.0010 (12)
C24	0.0290 (15)	0.0284 (17)	0.0211 (14)	−0.0083 (13)	0.0064 (12)	−0.0009 (12)
C25	0.0241 (14)	0.0214 (15)	0.0194 (13)	−0.0051 (12)	0.0063 (11)	−0.0009 (11)
C26	0.0179 (13)	0.0209 (14)	0.0223 (13)	−0.0025 (12)	0.0054 (10)	0.0000 (11)
C27	0.0324 (16)	0.0277 (16)	0.0344 (16)	−0.0086 (14)	0.0133 (13)	0.0018 (14)
C28	0.0269 (15)	0.0346 (19)	0.0387 (17)	−0.0073 (14)	0.0114 (13)	−0.0130 (15)

### Geometric parameters (Å, °)

**Table:**

C1—C12	1.519 (4)	C15—C16	1.537 (3)
C1—C2	1.528 (4)	C15—C20	1.552 (4)
C1—C7	1.545 (4)	C16—H16A	0.9800
C1—H1A	1.0000	C16—H16B	0.9800
C2—C3	1.495 (4)	C16—H16C	0.9800
C2—H2A	0.9900	C17—C18	1.525 (3)
C2—H2B	0.9900	C17—H17A	1.0000
C3—C5	1.342 (4)	C18—C19	1.554 (4)
C3—C4	1.499 (4)	C18—H18A	0.9900
C4—H4A	0.9800	C18—H18B	0.9900
C4—H4B	0.9800	C19—C20	1.552 (4)
C4—H4C	0.9800	C19—H19A	0.9900
C5—C6	1.499 (4)	C19—H19B	0.9900
C5—H5A	0.9500	C20—C21	1.538 (4)
C6—C7	1.541 (3)	C20—H20A	1.0000
C6—H6A	0.9900	C21—C22	1.529 (4)
C6—H6B	0.9900	C21—C23	1.533 (4)
C7—C8	1.539 (3)	C21—H21A	1.0000
C7—C9	1.546 (4)	C22—H22A	0.9800
C8—H8A	0.9800	C22—H22B	0.9800
C8—H8B	0.9800	C22—H22C	0.9800
C8—H8C	0.9800	C23—C24	1.527 (4)
C9—C10	1.538 (4)	C23—H23A	0.9900
C9—C13	1.542 (4)	C23—H23B	0.9900
C9—H9A	1.0000	C24—C25	1.524 (4)
C10—C17	1.520 (4)	C24—H24A	0.9900
C10—C11	1.521 (4)	C24—H24B	0.9900
C10—H10A	1.0000	C25—C26	1.527 (4)
C11—C12	1.520 (4)	C25—H25A	0.9900
C11—H11A	0.9900	C25—H25B	0.9900
C11—H11B	0.9900	C26—C28	1.518 (4)
C12—H12A	0.9900	C26—C27	1.528 (4)
C12—H12B	0.9900	C26—H26A	1.0000
C13—C14	1.534 (4)	C27—H27A	0.9800
C13—H13A	0.9900	C27—H27B	0.9800
C13—H13B	0.9900	C27—H27C	0.9800
C14—C15	1.529 (3)	C28—H28A	0.9800
C14—H14A	0.9900	C28—H28B	0.9800
C14—H14B	0.9900	C28—H28C	0.9800
C15—C17	1.537 (4)		
			
C12—C1—C2	111.0 (2)	C14—C15—C20	116.5 (2)
C12—C1—C7	113.1 (2)	C17—C15—C20	100.38 (19)
C2—C1—C7	112.1 (2)	C16—C15—C20	109.9 (2)
C12—C1—H1A	106.7	C15—C16—H16A	109.5
C2—C1—H1A	106.7	C15—C16—H16B	109.5
C7—C1—H1A	106.7	H16A—C16—H16B	109.5
C3—C2—C1	113.0 (2)	C15—C16—H16C	109.5
C3—C2—H2A	109.0	H16A—C16—H16C	109.5
C1—C2—H2A	109.0	H16B—C16—H16C	109.5
C3—C2—H2B	109.0	C10—C17—C18	118.5 (2)
C1—C2—H2B	109.0	C10—C17—C15	115.10 (19)
H2A—C2—H2B	107.8	C18—C17—C15	104.3 (2)
C5—C3—C4	122.7 (3)	C10—C17—H17A	106.0
C5—C3—C2	121.1 (3)	C18—C17—H17A	106.0
C4—C3—C2	116.2 (3)	C15—C17—H17A	106.0
C3—C4—H4A	109.5	C17—C18—C19	102.6 (2)
C3—C4—H4B	109.5	C17—C18—H18A	111.2
H4A—C4—H4B	109.5	C19—C18—H18A	111.2
C3—C4—H4C	109.5	C17—C18—H18B	111.2
H4A—C4—H4C	109.5	C19—C18—H18B	111.2
H4B—C4—H4C	109.5	H18A—C18—H18B	109.2
C3—C5—C6	124.2 (2)	C20—C19—C18	107.4 (2)
C3—C5—H5A	117.9	C20—C19—H19A	110.2
C6—C5—H5A	117.9	C18—C19—H19A	110.2
C5—C6—C7	114.1 (2)	C20—C19—H19B	110.2
C5—C6—H6A	108.7	C18—C19—H19B	110.2
C7—C6—H6A	108.7	H19A—C19—H19B	108.5
C5—C6—H6B	108.7	C21—C20—C19	111.5 (2)
C7—C6—H6B	108.7	C21—C20—C15	119.2 (2)
H6A—C6—H6B	107.6	C19—C20—C15	103.5 (2)
C8—C7—C6	108.1 (2)	C21—C20—H20A	107.4
C8—C7—C1	110.9 (2)	C19—C20—H20A	107.4
C6—C7—C1	106.8 (2)	C15—C20—H20A	107.4
C8—C7—C9	111.8 (2)	C22—C21—C20	113.8 (2)
C6—C7—C9	110.2 (2)	C22—C21—C23	110.4 (2)
C1—C7—C9	108.90 (19)	C20—C21—C23	110.3 (2)
C7—C8—H8A	109.5	C22—C21—H21A	107.3
C7—C8—H8B	109.5	C20—C21—H21A	107.3
H8A—C8—H8B	109.5	C23—C21—H21A	107.3
C7—C8—H8C	109.5	C21—C22—H22A	109.5
H8A—C8—H8C	109.5	C21—C22—H22B	109.5
H8B—C8—H8C	109.5	H22A—C22—H22B	109.5
C10—C9—C13	111.1 (2)	C21—C22—H22C	109.5
C10—C9—C7	113.4 (2)	H22A—C22—H22C	109.5
C13—C9—C7	114.1 (2)	H22B—C22—H22C	109.5
C10—C9—H9A	105.8	C24—C23—C21	114.9 (2)
C13—C9—H9A	105.8	C24—C23—H23A	108.5
C7—C9—H9A	105.8	C21—C23—H23A	108.5
C17—C10—C11	111.6 (2)	C24—C23—H23B	108.5
C17—C10—C9	109.1 (2)	C21—C23—H23B	108.5
C11—C10—C9	110.6 (2)	H23A—C23—H23B	107.5
C17—C10—H10A	108.5	C25—C24—C23	112.0 (2)
C11—C10—H10A	108.5	C25—C24—H24A	109.2
C9—C10—H10A	108.5	C23—C24—H24A	109.2
C12—C11—C10	111.7 (2)	C25—C24—H24B	109.2
C12—C11—H11A	109.3	C23—C24—H24B	109.2
C10—C11—H11A	109.3	H24A—C24—H24B	107.9
C12—C11—H11B	109.3	C24—C25—C26	116.0 (2)
C10—C11—H11B	109.3	C24—C25—H25A	108.3
H11A—C11—H11B	107.9	C26—C25—H25A	108.3
C1—C12—C11	111.2 (2)	C24—C25—H25B	108.3
C1—C12—H12A	109.4	C26—C25—H25B	108.3
C11—C12—H12A	109.4	H25A—C25—H25B	107.4
C1—C12—H12B	109.4	C28—C26—C27	110.6 (2)
C11—C12—H12B	109.4	C28—C26—C25	112.1 (2)
H12A—C12—H12B	108.0	C27—C26—C25	109.4 (2)
C14—C13—C9	113.1 (2)	C28—C26—H26A	108.2
C14—C13—H13A	109.0	C27—C26—H26A	108.2
C9—C13—H13A	109.0	C25—C26—H26A	108.2
C14—C13—H13B	109.0	C26—C27—H27A	109.5
C9—C13—H13B	109.0	C26—C27—H27B	109.5
H13A—C13—H13B	107.8	H27A—C27—H27B	109.5
C15—C14—C13	111.2 (2)	C26—C27—H27C	109.5
C15—C14—H14A	109.4	H27A—C27—H27C	109.5
C13—C14—H14A	109.4	H27B—C27—H27C	109.5
C15—C14—H14B	109.4	C26—C28—H28A	109.5
C13—C14—H14B	109.4	C26—C28—H28B	109.5
H14A—C14—H14B	108.0	H28A—C28—H28B	109.5
C14—C15—C17	107.4 (2)	C26—C28—H28C	109.5
C14—C15—C16	110.4 (2)	H28A—C28—H28C	109.5
C17—C15—C16	112.0 (2)	H28B—C28—H28C	109.5
			
C12—C1—C2—C3	174.5 (2)	C13—C14—C15—C17	55.7 (3)
C7—C1—C2—C3	46.9 (3)	C13—C14—C15—C16	−66.6 (3)
C1—C2—C3—C5	−14.8 (4)	C13—C14—C15—C20	167.2 (2)
C1—C2—C3—C4	166.6 (2)	C11—C10—C17—C18	−55.8 (3)
C4—C3—C5—C6	177.6 (3)	C9—C10—C17—C18	−178.2 (2)
C2—C3—C5—C6	−1.0 (4)	C11—C10—C17—C15	179.9 (2)
C3—C5—C6—C7	−15.5 (4)	C9—C10—C17—C15	57.4 (3)
C5—C6—C7—C8	−74.9 (3)	C14—C15—C17—C10	−58.9 (3)
C5—C6—C7—C1	44.6 (3)	C16—C15—C17—C10	62.4 (3)
C5—C6—C7—C9	162.8 (2)	C20—C15—C17—C10	178.9 (2)
C12—C1—C7—C8	−70.1 (3)	C14—C15—C17—C18	169.6 (2)
C2—C1—C7—C8	56.4 (3)	C16—C15—C17—C18	−69.1 (3)
C12—C1—C7—C6	172.3 (2)	C20—C15—C17—C18	47.4 (2)
C2—C1—C7—C6	−61.1 (3)	C10—C17—C18—C19	−166.5 (2)
C12—C1—C7—C9	53.3 (3)	C15—C17—C18—C19	−37.0 (2)
C2—C1—C7—C9	179.8 (2)	C17—C18—C19—C20	12.3 (3)
C8—C7—C9—C10	70.0 (3)	C18—C19—C20—C21	145.7 (2)
C6—C7—C9—C10	−169.8 (2)	C18—C19—C20—C15	16.4 (3)
C1—C7—C9—C10	−52.9 (3)	C14—C15—C20—C21	81.9 (3)
C8—C7—C9—C13	−58.6 (3)	C17—C15—C20—C21	−162.6 (2)
C6—C7—C9—C13	61.6 (3)	C16—C15—C20—C21	−44.5 (3)
C1—C7—C9—C13	178.5 (2)	C14—C15—C20—C19	−153.6 (2)
C13—C9—C10—C17	−52.1 (3)	C17—C15—C20—C19	−38.1 (2)
C7—C9—C10—C17	177.8 (2)	C16—C15—C20—C19	79.9 (3)
C13—C9—C10—C11	−175.1 (2)	C19—C20—C21—C22	−175.7 (2)
C7—C9—C10—C11	54.7 (3)	C15—C20—C21—C22	−55.2 (3)
C17—C10—C11—C12	−176.7 (2)	C19—C20—C21—C23	59.6 (3)
C9—C10—C11—C12	−55.2 (3)	C15—C20—C21—C23	−180.0 (2)
C2—C1—C12—C11	177.1 (2)	C22—C21—C23—C24	76.7 (3)
C7—C1—C12—C11	−55.8 (3)	C20—C21—C23—C24	−156.7 (2)
C10—C11—C12—C1	56.1 (3)	C21—C23—C24—C25	−179.9 (2)
C10—C9—C13—C14	53.5 (3)	C23—C24—C25—C26	−172.8 (3)
C7—C9—C13—C14	−176.7 (2)	C24—C25—C26—C28	−62.8 (3)
C9—C13—C14—C15	−56.0 (3)	C24—C25—C26—C27	174.2 (2)
